# Alzheimer's disease in the *Plasticene* era: a clinicopathological update on the dual sequestration of amyloid and tau as hijacked innate immune responses

**DOI:** 10.17879/freeneuropathology-2026-9368

**Published:** 2026-06-22

**Authors:** Michael A. S. Guth

**Affiliations:** 1 Institute for Neuroplasticity Research, Oak Ridge, USA

**Keywords:** Alzheimer's disease, Amyloid-β, Tau, Nanoplastics, Neuroinflammation, Innate immunity, Pyroptosis, Glymphatic system, Neuropathology, Environmental toxicology

## Abstract

**Background:** The defining neuropathological hallmarks of Alzheimer's disease (AD)—amyloid-β (Aβ) plaques and tau neurofibrillary tangles (NFTs)—are now understood to exist along a continuum with brain aging, yet their fundamental trigger in sporadic disease remains enigmatic. The clinical failure of therapies targeting these proteins, despite their successful removal, underscores a critical dissociation between hallmark pathology and core pathogenesis.

**Objective:** This clinicopathological update synthesizes emerging evidence on pervasive environmental nanoplastics (NPs) with the persistent paradoxes of AD to propose the dual sequestration hypothesis (DSH).

**Methods and Results:** We suggest that Aβ plaques and tau NFTs could be reinterpreted as evolutionarily conserved, compartment-specific innate immune sequestration mechanisms—an extracellular "sarcophagus" and an intracellular "lockbox"—based on their roles in microbial defense and stress response. We posit that in the modern *"Plasticene"* era, indestructible NPs detected in human cerebrospinal fluid (CSF) and brain tissue act as permanent, inorganic nucleation seeds that hijack these responses, forming indigestible synthetic protein complexes. NPs directly nucleate Aβ fibrillation and tau hyperphosphorylation, initiating the sequestration response. Chronic microglial engagement with these complexes triggers a state of "immune frustration," leading to a maladaptive phase transition. This pivot could be explained by glutamate excitotoxicity, which drives microglial NLRP3 inflammasome activation and pyroptotic cell death. Lytic pyroptosis liberates intact synthetic seeds into the paravascular space, where they are distributed via glymphatic flow, physically obstructing clearance and providing a mechanistic model for the stereotypical progression of Braak stages.

**Conclusion:** The DSH offers a unified explanation for the therapeutic failure of anti-Aβ/anti-tau antibodies (which remove the biological response but not the synthetic trigger) and amyloid-related imaging abnormalities (ARIA) as an inflammatory rebound. It necessitates a paradigm shift in neuropathological practice, calling for novel detection techniques to visualize the synthetic core within classical lesions, thereby unifying environmental etiology with canonical pathology. The presence of synthetic NPs at the physical center of Aβ plaques and tau tangles in human AD brain tissue is currently a prediction of the DSH awaiting empirical validation, not an established finding.

## Key points

- Functional reframing: Aβ plaques and tau NFTs could be understood as evolutionarily conserved sequestration mechanisms—the extracellular "sarcophagus" and intracellular "lockbox"—designed to wall off toxic material (Soscia et al., 2010; Kumar et al., 2016).
- The synthetic trigger: Pervasive environmental NPs act as permanent, non-degradable nucleation seeds, hijacking innate immune containment to form indestructible protein-synthetic complexes (Gou et al., 2024; Gecegelen et al., 2025; He et al., 2025).
- The pyroptotic pivot: Disease progression could be driven by a maladaptive phase transition (Ferrer, 2022). Chronic "immune frustration" triggers a glutamate-mediated excitotoxic cascade, resulting in microglial NLRP3 inflammasome activation and lytic pyroptosis (Lassmann, 2022; Wang & Shen, 2024).
- Glymphatic failure and spread: Pyroptotic events liberate intact synthetic-protein complexes. These "seeds" follow glymphatic drainage pathways, physically obstructing fluid clearance and offering an explanation for the spatio-temporal progression of Braak stages (Iliff et al., 2012; Rasmussen et al., 2022).
- Clinical implications: Anti-amyloid and anti-tau therapies fail because they remove the biological containment while re-exposing the toxic synthetic core, which provides a mechanistic explanation for ARIA (Sweeney et al., 2018; Sperling et al., 2023). This incompleteness, not inherent "badness," explains their modest benefits and adverse effects.
- Methodological call to action: Standard histopathological processing (solvents, heat, embedding) may inadvertently dissolve or mask synthetic cores. A transition toward spectroscopy, pyrolysis-gas chromatography/mass spectrometry (py-GC/MS), and cryo-preservation is required to validate environmental toxicants within hallmark lesions (McKenzie et al., 2024).

## 1. Introduction: the converging crises in Alzheimer's disease

### 1.1. The clinicopathological impasse: dissociation of pathology from pathogenesis

Neuropathologically, Alzheimer's disease (AD) is defined by the coexistence of extracellular amyloid-β (Aβ) plaques and intraneuronal phosphorylated tau neurofibrillary tangles (NFTs) (Jellinger, 2020). For decades, the dominant "amyloid cascade hypothesis" assigned a causative, toxic role to Aβ, a framework that logically culminated in immunotherapies designed to clear plaques. The recent development of monoclonal antibodies (mAbs) such as lecanemab and donanemab has achieved this goal, demonstrating a significant reduction in cerebral amyloid burden. However, this success has revealed a profound and troubling dissociation: marked plaque clearance yields only modest, transient slowing of cognitive decline, not arrest or reversal (Budd Haeberlein et al., 2022; Plascencia-Villa & Perry, 2023; van Dyck et al., 2023). This "efficacy gap" signals a fundamental flaw in a pathogen-centered model of Aβ.

The magnitude of this impasse is underscored by the staggering historical rate of clinical attrition. Between 1998 and 2017 alone, there were approximately 98 unique compound failures in the development of AD therapeutics (Kim et al., 2022). This near-total failure rate (exceeding 99 % for disease-modifying agents) reflects a systemic inability of current models to address the core pathogenesis. These failures are not merely statistical anomalies. They point to a profound ontological error in drug discovery: targeting the biological 'sarcophagus' or 'lockbox' while ignoring the biological pathogen or indestructible synthetic trigger that provoked its formation in the first place.

Compounding this therapeutic failure is the high incidence of amyloid-related imaging abnormalities (ARIA), a dangerous side effect featuring vasogenic edema and microhemorrhages. ARIA is not a mere pharmacological artifact but a pathognomonic sign of an unresolved underlying process—a clue that dismantling plaques may unleash, rather than resolve, the true disease driver (Sweeney et al., 2018). Since 2010, more than 200 drugs proposed to treat Alzheimer's have either failed in clinical trials or were abandoned (Anderson et al. 2017; Atri, 2019). Similarly, the centrality of tau pathology, while unequivocally linked to neuronal loss and cognitive impairment, faces a similar explanatory crisis. Anti-tau therapies have likewise struggled to demonstrate clinical efficacy. The field is, thus, left with a core clinicopathological dilemma: the defining proteinaceous lesions are necessary for diagnosis, but their removal is insufficient for cure. This impasse demands a radical re-evaluation of the biological nature of Aβ and tau aggregates, moving beyond their role as mere endpoints of pathology (Walker, 2020).

### 1.2. The environmental insurgency: the brain in the *Plasticene* era

Parallel to this clinical quandary, environmental science has uncovered a novel, pervasive threat to neural integrity. Micro- and nanoplastics (NPs), ubiquitous contaminants of the *Anthropocene*, have infiltrated global ecosystems and, consequently, the human body. These synthetic polymer particles have been confirmed to breach critical biological barriers and have been detected in human blood, placenta, and, most critically, in cerebrospinal fluid (CSF) and brain parenchyma (Bhattacharyya et al., 2025; He et al., 2025; Lu et al., 2025; Nihart et al., 2025). Emerging epidemiological and toxicological evidence links their presence to neuroinflammation, cerebrovascular dysfunction, and an increased risk of dementia (Gecegelen et al., 2025; Chakrabarti, 2026; Wang et al., 2026). The modern brain is, therefore, chronically inundated with indestructible synthetic material on a scale unprecedented in human history, a period we term the *Plasticene* era.

The scale and urgency of this environmental threat have been recognized across disciplines. Thompson et al. (2024), in their retrospective marking of twenty years of microplastic pollution research, concluded that these particles now represent a "planetary boundary threat." Microplastics pose unknown long-term biological consequences, including neurological health. A comprehensive health impact assessment by Lamoree et al. (2025) identified the central nervous system (CNS) as a critical organ of concern. Micro- and nanoplastics can cross the blood-brain barrier (BBB), trigger neuroinflammation, and potentially accelerate protein aggregation warrants urgent investigation. The dual sequestration hypothesis (DSH) directly addresses these calls by proposing a specific mechanistic pathway linking plastic particulates to AD pathology.

This environmental insurgency coincides with a growing recognition in neurodegenerative disease (NDD) research of the role of exogenous exposures, shifting the etiological focus toward gene-environment interactions (Crary, 2024). The convergence of these two truths—clearing hallmark proteins does not cure AD and the modern brain is saturated with a novel class of biopersistent toxicants—forms the critical context for a new synthesis.

### 1.3. Thesis: the dual sequestration hypothesis as a clinicopathological synthesis

We propose the DSH as a unifying framework to resolve these converging crises. This clinicopathological update posits that sporadic AD, particularly in its modern manifestation, could be understood as a disease of maladaptive innate immunity. The DSH suggests reframing Aβ and tau pathologies not as intrinsic pathogens but as visible remnants of overwhelmed, evolutionarily conserved sequestration responses. The DSH does not deny that Aβ and tau can exert toxicity in excess or that alternative views regarding their primary pathogenicity have merit. Rather, it reframes their aggregation as an evolutionarily conserved containment response—one that becomes maladaptive when the brain faces an indestructible trigger for which no evolutionary precedent exists.

In this model, Aβ could be seen as an extracellular "sarcophagus," a first-responder mechanism that encloses pathogens or insoluble or toxic material in the interstitial space, a role supported by its antimicrobial and metal-chelating properties (Atwood et al., 1998; Soscia et al., 2010). Tau, in turn, could function as an intracellular "lockbox," attempting to isolate harmful material that has been internalized. These may represent protective, containment strategies. The catastrophic shift of the *Plasticene* era is the introduction of the indestructible synthetic polymer—NPs—which act as permanent, non-biodegradable nucleation seeds. These seeds hijack the ancient sequestration machinery, leading to the formation of permanent, enzymatically indigestible "synthetic-protein complexes" (Gou et al., 2024).

The DSH contends that disease progression occurs via a maladaptive phase transition from stable containment to lytic failure (Ferrer, 2022). The chronic burden of these indigestible complexes leads to microglial "immune frustration," a metabolic and inflammatory tipping point. This state could be ignited by glutamate-mediated excitotoxicity, triggering microglial NLRP3 inflammasome activation and pyroptosis—a fiery, lytic cell death (Lassmann, 2022; Wang & Shen, 2024). Pyroptosis liberates the synthetic seeds, allowing them to propagate via the brain's glymphatic drainage system, mechanically obstructing flow and seeding pathology in a pattern that recapitulates Braak stages (Iliff et al., 2012; Rasmussen et al., 2022).

This framework offers a direct explanation for the therapeutic paradox: mAbs remove the proteinaceous sarcophagus but leave the synthetic splinter exposed, causing inflammatory rebound (ARIA) and continued seeding. It shifts the etiological focus from the host's response to the environmental trigger and the failure of the clearance systems meant to handle it. By integrating planetary-scale environmental change with molecular neuropathology, the DSH moves the field beyond the amyloid-tau cul-de-sac, offering a new mechanistic narrative for diagnosis, therapeutic strategy, and prevention.

### 1.4. The historical gradient of triggers: from industrial contaminants to the *Plasticene*

A complete understanding of the DSH requires situating the *Plasticene* trigger within a broader historical timeline of escalating environmental neural incursion. AD was not absent before the modern era—historical cases exist—but its incidence has risen dramatically over the past century in a pattern that cannot be explained by aging alone. In the 2024 Alzheimer's Association facts and figures report, deaths from AD increased more than 140 % between 2000 and 2021, while deaths from stroke, heart disease, and human immunodeficiency virus (HIV) decreased. The DSH proposes that different eras introduced qualitatively distinct triggers, each capable of hijacking the Aβ/tau sequestration machinery, with the *Plasticene* representing the most potent and persistent challenge:

- Pre-Industrial era (prior to 1880): Low baseline AD incidence. Triggers were primarily genetic (autosomal dominant mutations, APOE4), age-related proteostatic decline, and occasional pathogen-mediated sequestration events. AD existed but was comparatively rare relative to the modern epidemic.
- Industrial Revolution (1880–1910): Rapid industrialization introduced widespread occupational and environmental exposure to industrial solvents (benzene, toluene, trichloroethylene), heavy metals (lead, mercury, cadmium), and coal combustion particulates. These agents are established neurotoxicants and, within the DSH framework, would have acted as non-degradable or slowly cleared nucleation seeds. Notably, this period precedes the widespread introduction of processed foods and plastics, yet likely contributed to the early 20th-century rise in dementia prevalence.
- Progressive era / World War I (1910–1940): The mass production of canned foods introduced new vectors for contamination: tin, lead solder (leaching into acidic foods such as tomatoes), and early synthetic preservatives. The establishment of the U.S. Department of Agriculture's food inspection apparatus—systematically understaffed from its inception to the present day—meant that regulatory oversight failed to keep pace with industrial food production. The combination of occupational, environmental, and dietary contaminant and heavy metal exposure created a sustained "industrial load" on neural resilience. European food safety faced analogous challenges compounded by cross-border trade and inconsistent inspection standards.
- Mid-century cumulative burden (1940–1975): Exponential growth in chemical manufacturing introduced novel synthetic compounds (organophosphates, polychlorinated biphenyls, dioxins, phthalates) across environmental compartments. Notably, this period also saw the proliferation of synthetic food additives, preservatives, and packaging materials. The DSH posits that these decades represent a transitional phase in which multiple, potentially synergistic triggers accumulated, setting the stage for the rise in AD incidence observed in late-20th-century epidemiological data.
- The *Plasticene* era (1975–present): The exponential rise in global plastic production and waste introduced a trigger for which evolution had no precedent: a particulate, topologically complex, and enzymatically indestructible foreign body. Unlike earlier threats, a nanoplastic seed, once sequestered by Aβ, cannot be degraded. The sarcophagus becomes a permanent tomb. This era coincides with the most dramatic increase in AD incidence, disability-adjusted life years, and public health burden.

The DSH does not claim that AD did not exist before the *Plasticene* era. Rather, we propose that the historical trajectory of AD incidence reflects the sequential introduction and accumulation of novel environmental triggers, each capable of overwhelming the brain's sequestration machinery, with the indestructible NPs of the modern era representing the most potent and persistent challenge yet encountered. In *pre-Plasticene* populations with low industrial exposure, other triggers—heavy metals, chronic infections, traumatic brain injury, or genetic mutations—could similarly overwhelm the sequestration response, but at lower frequency and with different population incidence. The regulatory failures that permitted this escalating exposure—from understaffed food inspections to inadequate oversight of industrial chemicals—represent a systemic public health vulnerability that the DSH brings into sharp focus.

## 2. Functional reframing: Aβ and tau as hijacked innate immune responses

For decades, Aβ and tau were seen as toxic metabolic byproducts or inherent proteinopathies, a view reinforced by the dominant "amyloid cascade hypothesis." However, the significant disconnect observed in recent clinical trials—where substantial clearance of Aβ plaques and tau pathology results in only modest, temporary slowing of cognitive decline—calls for a radical re-examination (Plascencia-Villa & Perry, 2023; van Dyck et al., 2023). This therapeutic deadlock suggests that these key proteins might actually be the visible traces of an overwhelmed, evolutionarily conserved innate immune system.

### 2.1 The extracellular sarcophagus: amyloid-β

We suggest reframing the Aβ plaque not as a pathogenic endpoint but as an extracellular sarcophagus—a first-responder mechanism that sequesters insoluble or toxic material in the interstitial space. This view is supported by substantial evidence challenging Aβ's role as merely metabolic waste. From an evolutionary perspective, Aβ shows characteristics of a danger-precipitating protein. It exhibits potent, broad-spectrum anti-microbial activity in vitro and in model organisms, functioning as an antimicrobial peptide (Soscia et al., 2010; Kumar et al., 2016). Additionally, Aβ acts as a redox-active metal chelator, sequestering ions such as copper and iron to prevent Fenton chemistry and oxidative damage (Atwood et al., 1998). It is strongly upregulated in response to acute brain insults like infection and trauma, functioning as an acute-phase reactant essential for neuronal survival (Plant et al., 2003; Zuroff et al., 2017).

Oligomerization and fibrillization of Aβ physically execute this defensive function. Under the DSH, plaque formation could be a protective sequestration event—an attempt to "sarcophagus" a threat that cannot be enzymatically degraded or expelled from the immunologically privileged CNS. The heterogeneous morphology of plaques, ranging from diffuse to dense-core "neuritic" forms (Walker, 2020), may reflect the nature, chronicity, and indigestibility of the contained material. As with any immune response, this sequestration carries costs—chronic inflammation, metabolic drain, and collateral damage—that become catastrophic when the inciting agent cannot be degraded or expelled. Thus, describing this response as "adaptive" does not imply it is always successful or harmless.

**Figure 1: The sequestration paradox: biological vs. synthetic incursion:**
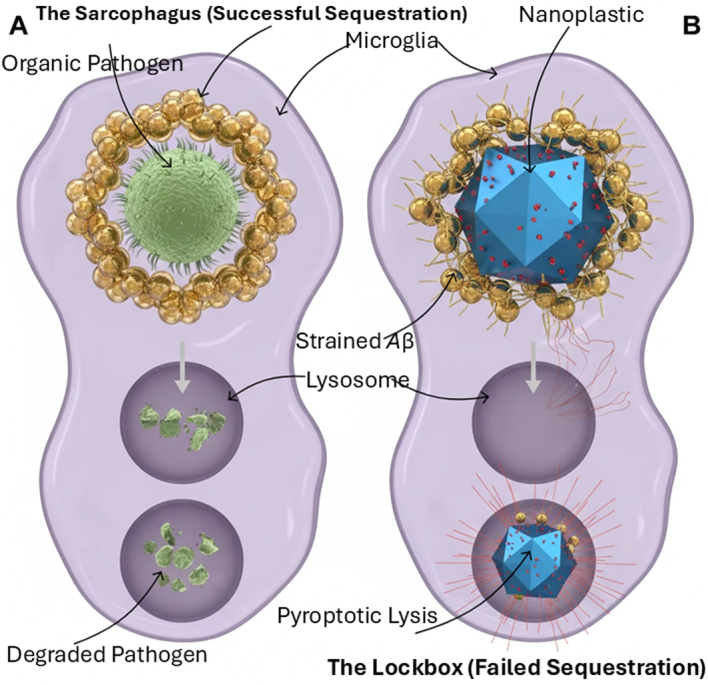
A comparative schematic of the microglial response to degradable versus non-degradable biological stressors. (**A**) The "sarcophagus" mechanism: microglia successfully encapsulate and enzymatically digest an organic pathogen like bacterium. The Aβ peptide forms a symmetrical, protective shell (gold spheres) around the pathogen within the phagolysosome, leading to complete degradation and metabolic resolution. (**B**) The "lockbox" failure: in the presence of a non-degradable, industrial nanoplastic core (metallic-blue icosahedron), Aβ sequestration occurs but fails to achieve enzymatic digestion. The persistence of the synthetic core leads to chronic lysosomal strain and the formation of a permanent, metabolic "dead-weight" within the microglial cytoplasm.

In the *pre-Plasticene* brain, this mechanism often succeeded against biological or ionic threats, such as pathogens or heavy metals, that could be chelated or slowly cleared (Bakulski et al., 2020). The catastrophic shift of the *Plasticene* era is the introduction of indestructible microplastics and NPs. These synthetic polymers act as permanent, non-biodegradable nucleation seeds that hijack this ancient response (Gou et al., 2024; Gecegelen et al., 2025). The Aβ sarcophagus, now built around an inorganic core, becomes a permanent inflammatory tomb—transforming a potentially adaptive defense into the cornerstone of pathology (**Figure 1**).

### 2.2 The intracellular lockbox: hyperphosphorylated tau

While Aβ patrols the extracellular space, the microtubule-associated protein tau functions within neurons. In its normal state, tau stabilizes micro-tubules, which are crucial for axonal transport and structural integrity. Its pathological transformation into hyperphosphorylated, aggregated NFTs is another diagnostic hallmark of AD and strongly correlates with cognitive decline (Jellinger, 2020).

**Figure 2: The glutamate detonator: transition from metabolic failure to pyroptosis:**
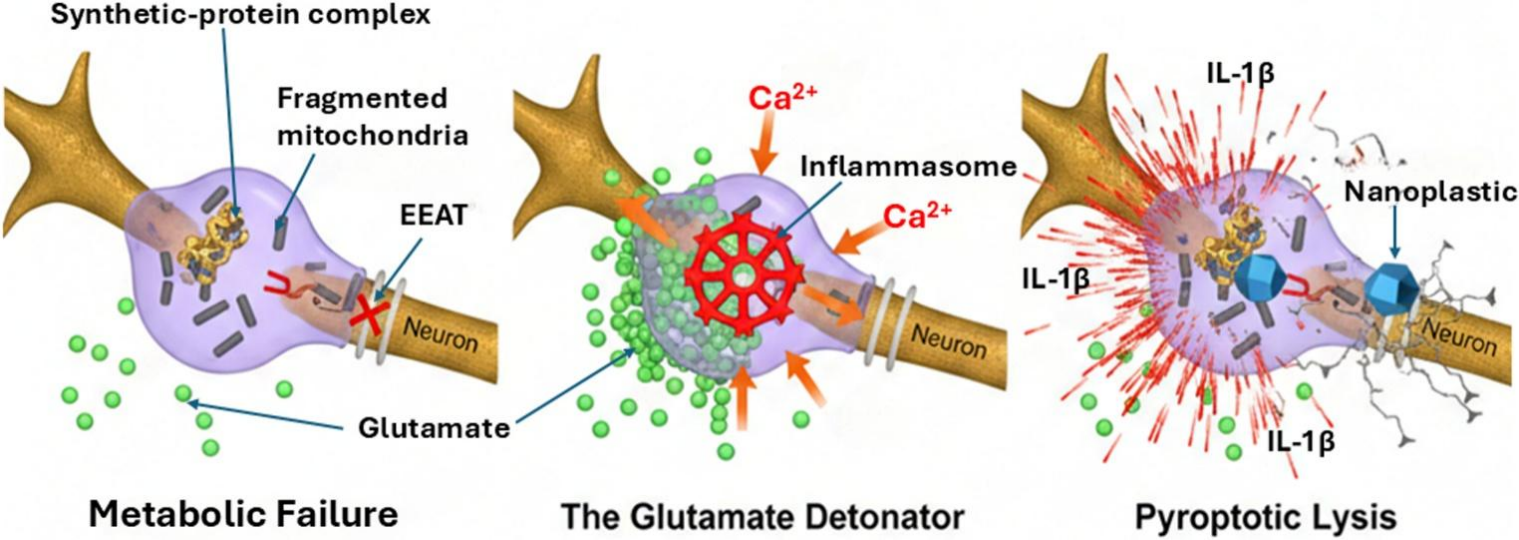
The three-stage cellular cascade leading to neurotoxic liberation (from left to right). Metabolic failure: accumulation of the synthetic-protein complex disrupts mitochondrial integrity (fragmented dark structures), leading to ATP depletion and the failure of excitatory amino acid transporters (indicated by red "X"). The glutamate detonator: extracellular glutamate (green spheres) pools at the synaptic interface, triggering a massive calcium (Ca²⁺) influx into the microglial cell. This secondary signal initiates the assembly of the NLRP3 inflammasome (red crystalline wheel). Pyroptotic lysis: the microglial cell undergoes inflammatory programmed cell death (pyroptosis), resulting in membrane rupture and the liberation of the intact, toxic nanoplastic core into the neuropil, accompanied by a surge of pro-inflammatory cytokines (IL-1β).

The DSH suggests viewing pathological tau aggregation as a parallel, intracellular sequestration strategy—the "internal lockbox." When toxic substances—such as misfolded proteins, damaged organelles, or, importantly, internalized foreign particles like NPs—breach the neuronal membrane and overwhelm degradation systems, the cell may resort to a drastic containment measure. The hyperphosphorylation and aggregation of tau into insoluble filaments could be an attempt to form an "internal lockbox", trapping the toxic cargo within the neuronal cytoplasm. This sequestration comes with a severe cost: the loss of tau's ability to stabilize microtubules disrupts axonal transport, leading to synaptic dysfunction and, ultimately, neuronal death (Jellinger, 2020). The tangle becomes a tombstone for a neuron that sacrificed its functional integrity in a failed containment effort (**Figure 2**).

### 2.3 The pathophysiological "seesaw": AD, cancer, and the innate immune trade-off

This dual-layer, compartmentalized sequestration logic provides a compelling framework for understanding the well-documented inverse epidemiological relationship between AD and cancer. The two diseases seem to occupy opposite ends of a fundamental biological spectrum governed by innate immune and cellular homeostatic mechanisms.

Cancer indicates a failure in cell sequestration and suppressed cell death, characterized by evading apoptotic signals, uncontrolled cell growth, and often the inactivation of tumor suppressors like p53. AD, especially in its environmental incursion (EI-AD) form, signifies immune hypersensitivity and excessive neuronal death. The brain's innate systems—the sarcophagus and lockbox—are continually activated by indestructible NPs. The constant reactivation results in persistent "immune frustration," a pyroptotic cascade, and a harmful acceleration of neuronal sequestration and loss.

Thus, the inverse relationship may reflect a fundamental trade-off in cellular resource allocation and risk management: an organism whose systems are geared toward aggressive containment and clearance of foreign or misfolded material (favoring an AD-type pathology) might be protected from the uncontrolled cellular growth that defines cancer, and vice versa. This "seesaw" reinforces the DSH's core premise: AD pathology could represent the maladaptive endpoint of normally protective, evolutionarily conserved innate immune responses pushed to failure by a new, permanent environmental trigger.

## 3. The novel trigger: NPs as permanent nucleation seeds

If Aβ and tau aggregates represent a conserved sequestration response, the central etiological question for sporadic AD shifts from "why do these proteins aggregate?" to "what is being sequestered?". The DSH identifies the defining environmental trigger of the modern AD epidemic:

synthetic micro- and NPs. These particulate pollutants represent a qualitative leap in the nature of neural insults—a permanent, indestructible nucleation seed that catastrophically hijacks the brain's containment mechanisms.

### 3.1. Physicochemical properties of a perfect pathological seed

NPs, typically defined as polymer particles less than 1000 nm in diameter, possess a unique constellation of properties that make them potent neurotoxicants and ideal pathological catalysts.

- Indestructibility: Unlike historical biological pathogens or organic chemicals, the carbon-carbon backbone of common polymers such as polyethylene, polypropylene, and polystyrene resists enzymatic degradation by mammalian enzymes, although research into engineered enzymatic breakdown via PETases is an active area of investigation (Cui et al., 2024). These polymers are non-biodegradable within the context of human physiology, creating a permanent foreign body. This biopersistence is worsened by the "Trojan Horse" effect, in which the lipophilic surfaces of NPs adsorb and concentrate environmental co-pollutants like heavy metals and persistent organic pollutants (Rochman et al., 2013), potentially aiding their co-translocation from the systemic circulation to the brain (Wang et al., 2026).
- Hydrophobicity and high-energy surfaces: NPs are often highly hydrophobic, creating high-energy interfaces in the aqueous biological milieu. These interfaces serve as potent inorganic scaffolds, dramatically lowering the kinetic and thermodynamic barriers to protein misfolding and aggregation.
- Toxicant vectors: Due to their lipophilic surfaces and large surface-area-to-volume ratio, NPs act as efficient vectors for a "Trojan horse" effect, thereby creating concentrated "toxic hubs" (Rochman et al., 2013; Brennecke et al., 2016). A comprehensive 2025 review by Gecegelen et al. concluded that microplastics demonstrate an "ability to cross the BBB and initiate several pathogenic pathways", including oxidative stress, neuroinflammation, and accelerated Aβ pathology through enhanced Aβ40 and Aβ42 nucleation.
- CNS penetration: Their nanoscale size allows NPs to bypass biological barriers. NPs have been detected crossing the BBB in vitro and in vivo, and, critically, have now been confirmed in human cerebrospinal fluid and post-mortem brain tissue, including the frontal cortex and deep structures (Bhattacharyya et al., 2025; He et al., 2025; Lu et al., 2025; Nihart et al., 2025). Their entry may be facilitated by systemic inflammation, compromised barrier integrity (Sweeney et al., 2018), or translocation along olfactory or trigeminal nerves, as with airborne particulate matter (Amato-Lourenço et al., 2024). This olfactory route is particularly relevant to the DSH, as the olfactory bulb has direct anatomical projections to the entorhinal cortex—a region that exhibits earliest tau pathology in Braak staging. Detection of microplastics in human olfactory bulb tissue (Amato-Lourenço et al., 2024) thus provides a plausible entry pathway connecting environmental exposure to the stereotypical spatiotemporal progression of AD pathology.

### 3.2. The inorganic scaffold: nucleating the sequestration response

The pathogenic process begins at the nanoscale interface between polymer and protein. In vitro studies consistently demonstrate that NP surfaces act as potent catalysts for Aβ fibrillation, accelerating the formation of β-sheet-rich, Thioflavin-T-positive aggregates (Gou et al., 2024; Gecegelen et al., 2025). The hydrophobic surface of polystyrene facilitates the interaction of hydrophobic fragments between Aβ monomers, which is the mechanistic driver of the "templating effect" and accelerated nucleation. (Gou et al., 2024).

This nucleation is profoundly amplified in vivo by the formation of a bio-corona. Upon entering a biological fluid, NPs are rapidly coated by a layer of host proteins, lipids, and other biomolecules (Monopoli et al., 2012; Tenzer et al., 2013). The composition of this corona defines the particle's biological identity. In the context of the DSH, the Aβ "sarcophagus" response may, in part, be an aggressive attempt to sequester an NP already shrouded in a corona of misfolded or damage-associated host proteins—a secondary containment of an already pathological complex.

The result is the formation of a synthetic-amyloid complex: an indestructible plastic core enveloped by a shell of host-derived proteins, primarily Aβ. This complex exhibits emergent toxicity. Recent work shows that such synthetic-protein complexes can inhibit key cellular phosphatases like PP1, PP2A, directly disrupting phosphorylation homeostasis—a finding that links NP exposure to the hyperphosphorylation of tau (Nadais et al., 2025). Supporting this mechanistic link, emerging in vitro evidence from a preprint indicates that sublethal doses of microplastics are sufficient to promote amyloid misfolding and acute metabolic impairment in cellular Alzheimer's disease models, highlighting the potent bioactivity of these synthetic-protein complexes (da Silva et al., 2025). The toxicity is not merely from bulk aggregation but from the specific poisoning of fundamental signaling machinery, which may also contribute to the bioenergetic crisis seen in AD (Butterfield & Halliwell, 2019; Wang et al., 2020).

### 3.3. From particulate exposure to persistent pathology: the *Plasticene* timeline

The DSH places this new trigger within a historical timeline of increasing industrial neural intrusion, explaining the rising rates of AD in the 20th and 21st centuries (see Section 1.4). Detecting microplastics/NPs in human brain tissue is no longer speculative; it is now an established finding (He et al., 2025; Nihart et al., 2025). Oxidative stress and the oxidative stress-sensitive TRPM2 channel are key in mediating multiple molecular and cellular changes that underlie AD-related cognitive decline (Wang, Wei et al., 2020). The "*Plasticene* era" thus introduces the ancient Aβ and tau sequestration systems to their ultimate, unwinnable challenge. The hijacking of this protective response by a permanent synthetic seed turns a potentially helpful defense into a chronic, self-sustaining disease process—the maladaptive phase transition at the heart of the DSH.

We recognize an important caveat: most experimental studies use nanoplastic concentrations far exceeding those yet measured in human brain tissue. Whether the lower, chronic, mixed-polymer, multi-decade exposure typical of human environmental exposure can recapitulate the same mechanistic cascade as acute high-dose experimental models is an open question requiring further investigation. However, we note that bioaccumulation over decades—combined with the indestructible nature of these particles—may render cumulative lifetime burden functionally equivalent to higher acute concentrations. Direct comparative studies across populations with varying exposure profiles are urgently needed: coastal versus inland residents (differing microplastic burdens from seafood and aerosolized sea spray), military personnel with >10 years of service versus civilians (differing exposure to synthetic materials, flame retardants, burn pits, and occupational particulates), industrial versus developing nations, and plant-based versus meat-based diets (differing bioaccumulation through trophic transfer). The DSH generates specific, testable predictions about these comparisons.

## 4. The hijacking and phase transition to disease

The DSH posits that the formation of synthetic-protein complexes is not the disease endpoint but rather the catalyst for a maladaptive biological cascade. The transition from stable, localized containment to progressive neuroinflammation represents a critical phase transition—a concept that aligns with views of AD as a pathological acceleration of brain aging (Ferrer, 2022). This transition is driven by the indigestibility of the NP core, which converts a protective immune response into a state of chronic immune frustration, culminating in a lytic failure that propagates pathology.

### 4.1. Formation of the indigestible complex and onset of "immune frustration"

The initial sequestration of an NP seed by Aβ, or the internalization of NP-protein complexes by neurons, triggers tau aggregation, forming an entity the brain cannot degrade (Gou et al., 2024). Microglia engage in futile phagocytosis, internalizing a target lysosomal enzymes cannot digest (Hickman et al., 2018). The persistent synthetic core promotes lysosomal membrane permeabilization, leakage of cathepsins, and sustained activation of the NLRP3 inflammasome (Campden & Zhang, 2019). At this stage, engagement may remain sublytic, characterized by chronic low-grade cytokine release and reactive oxygen species production, which contribute to a smoldering inflammatory milieu (Plascencia-Villa & Perry, 2023).

This state is what we term immune frustration: microglia are chronically activated, metabolically burdened, and perpetually signaling danger yet cannot resolve the insult—a context-dependent detrimental role (Lassmann, 2022). The sustained energy demand contributes to regional bioenergetic deficits, exacerbating cerebral glucose hypo-metabolism and fostering cerebral insulin resistance, a state increasingly framed as type 3 diabetes (de la Monte & Wands, 2008; Atabi et al., 2025). This metabolic impairment depletes cellular ATP, depletes NAD+ reserves, and impairs insulin signaling, creating a state of functional neuronal starvation (Cunnane et al., 2020; Wang et al., 2020). The microglial response, now detrimental, inflicts collateral damage on synapses and neurons.

### 4.2. The glutamate detonator: converting containment to catastrophe

Chronic neuroinflammation, driven by the persistent presence of synthetic protein complexes, fundamentally dysregulates glutamate homeostasis. Activated microglia and astrocytes release a cocktail of pro-inflammatory cytokines, such as TNF-α, IL-1β, that impair astrocytic reuptake of synaptic glutamate by downregulating the excitatory amino acid transporter 2 (EAAT2/GLT-1) (Plascencia-Villa & Perry, 2023). However, this failure is not merely regulatory; it is energetically mandated. The bioenergetic crisis characteristic of the "type 3 diabetes" phenotype creates a fatal metabolic bottleneck.

Because high-affinity glutamate reuptake against its concentration gradient is strictly ATP-dependent, mitochondrial failure and glucose hypometabolism induced by nanoplastic toxicity (Wang et al., 2020) render astrocytic pumps physically incapable of maintaining synaptic clearance. This "energy-starved" microenvironment ensures that glutamate reuptake failure is not a transient glitch but a permanent structural deficit. The resulting synaptic glutamate spillover leads to the chronic, pathological stimulation of extrasynaptic NMDA receptors. This persistent activation triggers a massive, uncontrolled influx of calcium (Ca²⁺), which further poisons mitochondrial respiration and ignites the "glutamate detonator."

This excitotoxic event acts as the critical phase transition from smoldering frustration to systemic catastrophe. Neuronal distress signals, including the release of damage-associated molecular patterns (DAMPs) and ATP, act as secondary "danger" ligands for microglial receptors. Most critically, the excitotoxic Ca²⁺ surge into neighboring microglia can directly catalyze the full assembly and activation of the previously primed NLRP3 inflammasome (Garaschuk, 2021; Zhang et al., 2021). In this framework, glutamate-mediated excitotoxicity is the spark that converts chronic, sublytic immune frustration into an acute, lytic, and self-propagating immune activation, effectively "detonating" the containment structures the brain worked so hard to build. The genetic architecture of late-onset AD provides independent corroboration for the centrality of the glutamate detonator within the DSH.

ABCA7, the ATP-binding cassette transporter A7, is the second most significant genetic risk determinant for AD after APOE. Long regarded primarily as a lipid transporter, its mechanistic role in neurodegeneration has remained poorly defined. Górska et al. (2026) have now provided the first comprehensive description of how ABCA7 deficiency amplifies glutamatergic neurotoxicity. In AD mouse models, ABCA7 ablation exacerbated excitotoxic damage by reducing enzymatic degradation of glutamate and upregulating NMDA, AMPA, and GABA-A receptor subunits. Critically, this effect was mediated primarily through lipid interaction, connecting the metabolic disruptions associated with ABCA7 risk variants. These risk variants include impaired cholesterol efflux, lipid droplet accumulation, and reduced mitochondrial membrane potential (von Maydell et al., 2025). All those risks can cause the failure of glutamate homeostasis. The ABCA7-NLRP3 inflammasome axis (Santos-Garcia et al., 2025) provides the mechanical link between this genetic risk variant and the pyroptotic cascade central to the DSH.

Within the DSH framework, this is a significant finding: individuals carrying ABCA7 risk variants represent a genetically sensitized population whose glutamate detonator threshold is constitutively lowered. The bioenergetic and lipid transport deficits imposed by ABCA7 dysfunction act synergistically with the metabolic drain of chronic immune frustration from nanoplastic sequestration, ensuring that the excitotoxic tipping point is reached earlier, more severely, and at lower burdens of synthetic-protein complexes. ABCA7 status may thus be a key determinant of individual susceptibility within the EI-AD spectrum.

Similarly, the APOE4 allele—the strongest genetic risk for late-onset AD—exacerbates tau pathology through cholesterol-induced degradation of protein phosphatase 2A (PP2A) and impairs glymphatic clearance via disruption of perivascular AQP4 localization (Ding et al., 2025). APOE4 carriers also exhibit accelerated BBB breakdown, reduced pericyte coverage, and enhanced neuroinflammation in response to systemic inflammatory challenges. Within the DSH, APOE4 would therefore amplify every stage of the pathogenic cascade: increased NP entry due to barrier compromise, impaired clearance of synthetic seeds due to glymphatic dysfunction, enhanced tau pathology due to phosphatase inhibition, and exacerbated inflammatory responses to the indigestible core. This multilayered synergy between the APOE4 genotype and the NP trigger may explain why APOE4 carriers are disproportionately represented among EI-AD patients, while also accounting for the incomplete penetrance of the allele-sufficient NP burden may still be required.

### 4.3. Stress as a threshold-lowering susceptibility factor

A critical question facing any etiological model of AD is differential susceptibility: why do some individuals with apparently similar exposure histories develop dementia while others remain cognitively intact? The DSH proposes that chronic psychological stress—particularly financial stress, risk of housing loss, divorce, medical crisis, and caregiving burden—acts as a threshold-lowering facilitator that sensitizes the brain to NP-induced pathology.

The mechanistic basis for this interaction is well-grounded in established neurobiology:

1. BBB compromise: Chronic stress elevates circulating glucocorticoids, which have been shown to increase BBB permeability by downregulating tight junction proteins (claudin-5, occludin) and enhancing transcytosis. A more permeable BBB allows greater NP entry into the brain parenchyma per unit of environmental exposure, amplifying the effective dose.
2. Glymphatic impairment: Stress-induced sympathetic activation and altered cerebrovascular dynamics impair glymphatic clearance, reducing the brain's ability to remove both NPs and their proteinaceous sequestrate. Slower clearance means longer residence time for synthetic seeds, increasing cumulative damage.
3. Excitotoxic sensitization: Chronic stress primes glutamatergic systems, lowers the seizure threshold, and enhances NMDA receptor responsiveness. This primes the "glutamate detonator" (Section 4.2), meaning that a smaller excitotoxic trigger is sufficient to initiate pyroptotic cascades.
4. Neurogenic suppression: Stress inhibits hippocampal neurogenesis and reduces neural reserve, depleting the brain's capacity to compensate for ongoing neuronal loss and thereby accelerating the clinical expression of pathology.

Thus, two individuals with identical lifetime NP burden may have radically different outcomes: the individual experiencing chronic midlife stress develops EI-AD earlier and more severely; the unstressed individual remains cognitively normal or develops pathology much later, if at all. Crucially, stress alone—without sufficient NP burden—does not cause AD. This explains why not everyone who experiences a bitter divorce or prolonged financial hardship develops Alzheimer's: stress is a multiplier, not a sufficient cause. The DSH thus provides a mechanistic framework for understanding the well-documented but poorly explained epidemiological association between psychosocial stress and dementia risk, while resolving the question of differential susceptibility that has plagued purely protein-centric models.

### 4.4. The pyroptotic cascade and "seed" liberation

The full activation of the NLRP3 inflammasome catalyzes cleavage of gasdermin-D, forming pores in the plasma membrane and leading to pyroptosis, a highly inflammatory, lytic form of programmed cell death (Wang & Shen, 2024). The microglial cell swells, bursts, and releases its cytoplasmic contents. This pyroptotic event is not merely inflammatory; it is propagative. The lytic release includes a burst of pro-inflammatory cytokines, such as IL-1β and IL-18, that recruit more microglia, a flood of DAMPs that amplify sterile inflammation, and, crucially, the intact synthetic-protein complexes that were trapped inside phagolysosomes. These complexes are liberated back into the neuropil, physically unchanged. The original NP seed, now potentially fragmented or associated with more misfolded protein from the dead cell's contents, is re-released—mobile and bioavailable for a new round of the cycle.

### 4.5. Glymphatic spread and the "drainage basin" model of propagation

**Figure 3: Macro-scale propagation: glymphatic basin stasis and retrograde seeding:**
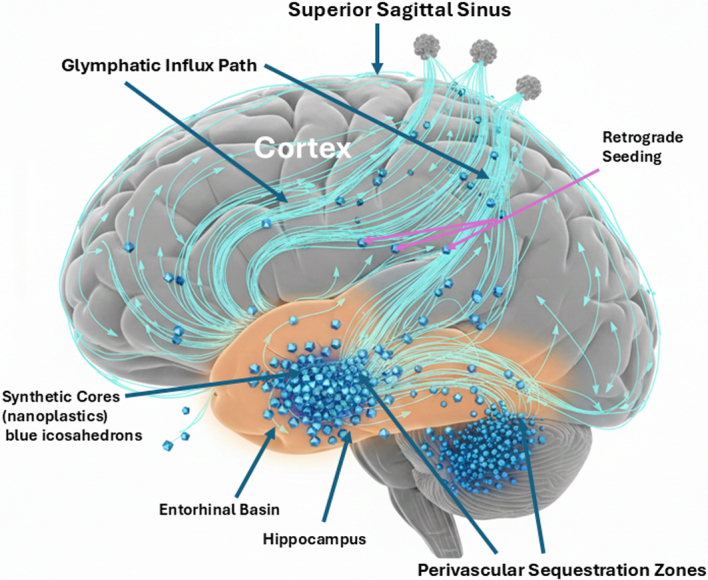
Fluid-dynamic mapping of particulate spread within the glymphatic system. In a sagittal cross-section of the human brain, the entorhinal cortex (amber glow) serves as the primary "drainage basin" where liberated synthetic cores (blue particulate) accumulate. High concentrations of these non-degradable particles in the perivascular sequestration zones induce "glymphatic stasis," clogging the perivascular clearance pathways (cyan streamlines). As the primary basin reaches capacity, retrograde seeding occurs, where particulate matter is carried by fluid dynamics into the hippocampus and neocortex, providing a mechanical explanation for the predictable anatomical progression of Braak stages.

The liberation of synthetic seeds into the interstitial fluid engages the brain's macroscopic waste-clearance system: the glymphatic pathway. This system facilitates exchange of cerebrospinal and interstitial fluids along perivascular spaces, driven by arterial pulsatility and dependent on astrocytic aquaporin-4 channels (Iliff et al., 2012; Rasmussen et al., 2022). The newly liberated NP-protein complexes can enter this paravascular flow. We propose they act as physical obstacles within these narrow channels. Their accumulation contributes to glymphatic stasis, impairing clearance of soluble Aβ, tau, and other metabolic waste, thereby compounding the toxic environment—a dysfunction linked to impaired aquaporin-4 function (Munk et al., 2023). Furthermore, this flow distributes the seeds (**Figure 3**).

This provides a powerful mechanistic model for the stereotypical progression of pathology. The initial sites of NP deposition and sequestration, such as regions with high perfusion or specific barrier vulnerabilities, become the first drainage basins where complexes form. Pyroptotic events in these regions release seeds into the local glymphatic flow, which then carries them to downstream, interconnected basins. This pattern of seed distribution via the brain's intrinsic plumbing system offers a direct explanation for the sequential progression of tau pathology described by Braak staging, moving from the transentorhinal and entorhinal cortex—an early drainage basin—into limbic and ultimately neocortical regions (Jellinger, 2020). In summary, the DSH mechanism is a self-perpetuating cycle: synthetic seed leads to sequestration, which leads to immune frustration, which is ignited by the glutamate detonator to cause pyroptotic lysis, resulting in seed liberation and subsequent glymphatic spread to establish new foci of sequestration. This cycle transforms a focal containment problem into a propagating system failure.

### 4.6. The choroid plexus (ChP): hydrodynamic bottleneck and ground zero

If the DSH is correct, the ChP should enlarge and become the sentinel hydrodynamic bottleneck as synthetic particles and their protein sequestrants accumulate. This terminal bottleneck or anatomical ground zero sets the stage for the DSH and systemic failure. Without ChP enlargement as the ultimate focal area for sequestration deposits, the glymphatic spread remains an unbounded process without a clear pathology. Conversely, if modern medicine cannot solve the sequestration and clogging problem at the ChP, then upstream treatments targeting the parenchyma will yield little or no clinical benefit.

The ChP stroma supports fenestrated (leaky) capillaries, distinct from the tight BBB, enabling molecular exchange between blood and CSF and transporting immune cells into the ventricles. With aging, the stroma can become hardened and fibrotic, accumulate calcium and iron deposits, and now the DSH predicts NP deposits as well. Non-degradable particles serve as a scaffold for age-related stromal hardening, thereby turning the ChP from a filter into a dam. When the ChP becomes a dam, upstream pressure—manifesting as increased intracranial pressure or glymphatic backflow—eventually overwhelms and kills the neurons. If the glymphatic backflow stops, the metabolic waste like Aβ and Tau remains inside the individual neurons, and they drown in their own metabolic waste. By integrating the enlarged ChP as a hydrodynamic sump, the DSH becomes a closed-loop system: trigger sequestration → lytic release → ChP clogging and enlargement.

An enlarged or hypertrophied ChP can overproduce CSF or physically obstruct its flow and thus cause the brain's ventricles to become enlarged or dilated (ventriculomegaly). Enlarged ventricles in the brain are a well-known hallmark of AD, yet their cause has remained mechanistically unexplained. The DSH provides that missing link: ventriculomegaly is the macroscopic consequence of microscopic clogging at the ChP.

Recent findings by Pang et al. (2026) provide clinical support for the DSH. They found that MRI scans of patients suffering from long COVID displayed enlarged ChP and reduced cerebral blood flow, which are both associated with AD-like dementia. Long COVID brain fog is an acute manifestation of the same sequestration phenomenon. The DSH is the unifying framework for both post-infectious (COVID) and environmental (NP) neurodegenerative risk. The clinicopathological cascade begins with ChP immune surveillance, followed by non-degradable particle trapping, barrier dysfunction, and glymphatic failure: a sequence now visible on medical imaging and traceable back to its molecular origins. At the ChP, viral or environmental toxicology crashes into clinical neurology.

The DSH defines the ChP as both the final stage of the accumulation and the starting point of the permanent dementia-like decline. With clinical urgency, the MRI findings of ChP enlargement are the definitive signal that the ground zero event is occurring in the patient. The ChP is where the sequestration cycle reaches critical mass, triggering the glymphatic failure that characterizes the transition from acute brain fog to chronic neurodegeneration. If the ChP is ground zero for the collapse, then a therapy targeting clearance of the ChP becomes the logical cure for this form of neurodegeneration.

## 5. Clinicopathological synthesis and implications

The DSH provides a coherent framework that clarifies the most perplexing clinicopathological paradoxes of AD. By reframing pathology as a failed response to an indestructible trigger, the model offers new interpretations of disease staging, therapeutic failure, and the definition of a disease subtype.

### 5.1. Reinterpreting Braak staging as a map of glymphatic seed distribution

The stereotypical progression of neurofibrillary tau pathology from the transentorhinal cortex through the limbic system and into the neocortex, formalized as Braak staging, is a cornerstone of AD neuropathology (Jellinger, 2020). Although often attributed to the prion-like transneuronal spread of pathological tau, this pattern also finds a powerful alternative explanation in the DSH. We propose that Braak staging reflect the anatomical flow of synthetic seeds via the brain's glymphatic drainage system. The entorhinal cortex, with its high degree of interconnectivity and role as a hub for fluid exchange, may serve as an initial drainage basin where circulating NPs first deposit and are sequestered—a hypothesis supported by the detection of microplastics in the human olfactory bulb, which has close anatomical connections to the entorhinal cortex (Amato-Lourenço et al., 2024). This olfactory bulb → entorhinal cortex pathway provides a plausible entry route that directly connects environmental inhalation of airborne microplastics to the earliest sites of tau pathology in Braak stage I/II, prior to neocortical spread.

The subsequent cycle of immune frustration, pyroptosis, and seed liberation at this site would inject synthetic-protein complexes into the local paravascular flow. This flow, guided by the brain's intrinsic architecture, would then distribute seeds to anatomically connected downstream regions—first to the hippocampus and limbic structures, and later to association cortices. This model posits that tau pathology follows the distribution of the synthetic trigger, not merely the self-propagation of tau alone. The glymphatic system, essential for clearance, ironically becomes a conduit for disease propagation when carrying indestructible cargo.

### 5.2. Explaining therapeutic failure and ARIA: the splinter analogy

The DSH provides a mechanistic rationale for the two most critical observations in recent AD clinical trials: the modest efficacy of amyloid-β-clearing antibodies and the high incidence of ARIA. Using a splinter analogy, the Aβ plaque is the inflamed, proteinaceous sarcophagus that forms around a foreign body—the synthetic splinter. Current mAbs such as lecanemab and donanemab are designed to dissolve the proteinaceous sarcophagus but lack a mechanism to remove or degrade the synthetic core (Plascencia-Villa & Perry, 2023; van Dyck et al., 2023).

**Figure 4: The ARIA paradox: iatrogenic de-shielding of toxic cores.**
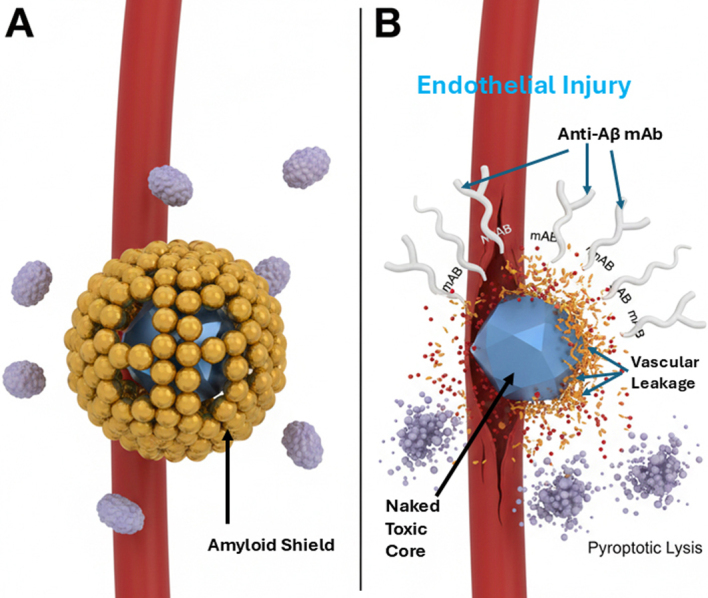
The clinical mechanism of ARIA under mAb therapy. (**A**) Native Sequestration: Prior to treatment, Aβ (gold) acts as a biological "sarcophagus" or "lead-shielding," sequestering the toxic synthetic core (blue) and protecting the cerebral vasculature from direct contact. (**B**) Antibody Intervention: Monoclonal antibodies (Y-shaped proteins) successfully bind to and dissolve the Aβ shell. However, this process "de-shields" the naked, indestructible nanoplastic core, allowing it to make direct contact with the vascular endothelium. The resulting mechanical friction induces endothelial injury (sky blue label), manifesting as vascular fraying, micro-hemorrhages (red droplets), and a localized inflammatory rebound. This provides a materials-science explanation for ARIA-E (edema) and ARIA-H (hemorrhage) as a consequence of removing the containment structure without neutralizing the underlying toxicant.

The therapeutic action of current mABs has two direct consequences. First, stripping away the Aβ shell re-exposes brain tissue to the concentrated cocktail of toxins adsorbed to the NP surface and presents the indigestible synthetic particle directly to microglia. This re-triggers the cycle of futile phagocytosis and NLRP3 inflammasome activation, igniting a diffuse inflammatory rebound. We emphasize that this does not mean anti-Aβ therapies are inherently "bad" or should be abandoned entirely. Rather, they are fundamentally incomplete: they remove the biological containment while leaving the indestructible synthetic core exposed. This incompleteness explains both their modest, transient benefits (in prodromal stages where total core burden is lower) and their adverse effects (ARIA). Second, NPs frequently accumulate in perivascular spaces. The pharmacological dissolution of the Aβ scaffold can destabilize the neurovascular unit at these sites of mechanical irritation, leading to increased vascular permeability (vasogenic edema, ARIA-E) and microhemorrhages (ARIA-H) (Sweeney et al., 2018) (**Figure 4**). Therefore, ARIA is not a mere side effect; it is the iatrogenic re-poisoning and structural weakening predicted by removing a containment structure without addressing the contained agent. An optimal therapeutic strategy would combine removal of the proteinaceous shell with simultaneous neutralization or extraction of the synthetic core.

The modest and transient clinical benefit observed in early-stage disease is also explained. In prodromal stages, the total burden of synthetic-core plaques is lower, and the associated bioenergetic drain is less severe. Removing the Aβ shell may temporarily reduce the inflammatory burden, providing a compromised system with a marginal functional reprieve (Sperling et al., 2023). However, once the chronic sequestration response has led to widespread mitochondrial failure, glymphatic clogging, and synaptic loss—the hallmarks of established dementia—merely clearing the protein is insufficient. This obstruction creates a localized "synaptic prison" in which the physical presence of synthetic-protein complexes prevents the clearance of glutamate and metabolic waste, transforming excitotoxicity from a transient event into a permanent state of the synaptic microenvironment.

The brain lacks the energetic and structural capacity to regenerate, a concept consistent with the view of AD as a systemic failure (Ferrer, 2022). Furthermore, the chronic inflammatory state exhausts the brain's regenerative potential by poisoning the stem and progenitor cell niche (van Velthoven & Goldman, 2019; Abbate 2023). This represents a pathological acceleration and perversion of the natural, age-related decline in hippocampal neurogenesis (Seib & Martin-Villalba, 2015), effectively crippling the brain's intrinsic capacity for cellular repair.

### 5.3. The role of intrinsic aging: substrate vs. driver

Critics of environmentally focused AD models often argue that aging is the primary driver, pointing to the strong correlation between advancing age and disease prevalence. The DSH does not dispute that aging is a powerful risk factor. However, several observations suggest that aging alone is insufficient as a primary causal explanation:

- Longevity without dementia: A substantial majority of individuals aged 85-101 do not develop AD. If aging were the primary driver, one would expect near-universal pathology at extreme ages, which is not observed.
- Rising incidence independent of demographics: Between 2000 and 2021, reported deaths from AD increased more than 140 %, while deaths from stroke, heart disease, and HIV decreased (2024 Alzheimer's Association facts and figures). This dramatic increase cannot be explained by population aging alone; it implicates environmental factors that have intensified over this period.
- Projected burden: Even with continued aging of the population, Alzheimer's is projected to affect approximately 13.8 million Americans by 2060—still a minority of the elderly population.

Within the DSH framework, we propose that intrinsic aging mechanisms create the vulnerable substrate upon which environmental triggers act, rather than serving as the primary driver themselves. These aging-related processes include:

- Proteostasis failure: Age-related decline in chaperone-mediated folding, ubiquitin-proteasome system efficiency, and autophagic flux reduces the brain's ability to handle misfolded proteins, whether endogenous or induced by NP exposure.
- DNA damage accumulation: Lifelong oxidative and replicative stress leads to genomic instability, compromising neuronal and glial resilience.
- Mitochondrial dysfunction: Age-related decline in oxidative phosphorylation, increased reactive oxygen species production, and reduced mitophagy create bioenergetic vulnerability.
- Steroid hormone decline: Age-related reductions in estrogen, testosterone, and DHEA eliminate neuroprotective signaling that maintains BBB integrity, supports synaptic plasticity, and modulates inflammatory tone.
- Glymphatic senescence: The brain's waste clearance system becomes less efficient with age, independent of exogenous triggers (Munk et al., 2023).

Thus, the DSH is best understood as an accelerationist model: aging creates the permissive background—declining proteostasis, impaired clearance, reduced regenerative capacity—upon which environmental triggers act. NPs (and historical triggers before them) convert the slow, naturally occurring age-related accumulation of protein aggregates into a rapidly progressive, inflammatory, self-propagating cascade. In the absence of significant NP exposure, aging alone can still produce AD pathology, but at lower rates, with longer latency, and often with slower progression. The *Plasticene* era has dramatically accelerated a process that already existed at low baseline incidence. The DSH therefore does not compete with aging-based models but rather explains why the age-associated pathology has become both more common and more aggressive in recent decades.

### 5.4. Defining a subtype: environmental incursion Alzheimer's Disease (EI-AD)

The paradigm shift from the amyloid cascade to the DSH necessitates the delineation of a major, modern clinicopathological subtype. The fundamental contrasts between the traditional model and the DSH are summarized in **Table 1**.

**Table 1:** Comparative framework of the amyloid cascade versus the dual sequestration hypothesis

**Feature**	**Amyloid cascade hypothesis**	**Dual sequestration hypothesis (DSH)**
Aβ plaque role	Primary pathogen: intrinsic proteinopathy	Protective sarcophagus: evolutionarily conserved innate immune sequestrant
Tau NFT role	Secondary toxicant: downstream of Aβ	Intracellular lockbox: last-resort containment of internalized/unresolved synthetic seeds
Primary trigger	Protein misfolding (stochastic/genetic)	Synthetic nanoplastics (environmental): indestructible inorganic nucleation seeds. The DSH recognizes that other triggers may predominate in some individuals or historical cohorts
Etiological focus	Endogenous protein toxicity	Incursion lifecycle: Failed host response to an exogenous, non-degradable agent
Braak staging	Prion-like transneuronal spread of tau	Glymphatic "drainage basin" flow: distribution of seeds via perivascular clearance dynamics
ARIA mechanism	Incidental off-target vascular inflammation	Iatrogenic re-poisoning: acute tissue re-exposure to the toxic core after shell dissolution
Therapeutic logic	Monolithic protein clearance	Multi-pronged Intervention: peripheral interception, core neutralization, and bioenergetic repair
Metabolic state	Secondary symptom of cell death	Type 3 diabetes phenotype bioenergetic failure driven by immune frustration and mitochondrial poisoning
Failure of mAbs	Temporal mismatch or selection of incorrect Aβ species	Containment dissolution: clearing the biological scaffold leaves the indestructible synthetic core
Neuropathology	Biological/histological (protein-centric)	Forensic/materials science: detection of synthetic polymer cores within biological lesions

This framework defines environmental incursion Alzheimer's disease (EI-AD). EI-AD is a progressive neurodegenerative disorder in which the primary etiological driver is the sequestration of non-biodegradable environmental particulates—primarily synthetic micro- and nanoplastics—within the central nervous system (He et al., 2025; Nihart et al., 2025). This trigger hijacks the Aβ/tau sequestration response, initiating a cascade of bioenergetic failure, chronic neuroinflammation, and synaptic dysfunction central to the DSH.

EI-AD is etiologically distinct from autosomal dominant AD (caused by APP/PSEN mutations) and may exist on a spectrum with, or accelerate, other genetic risk-associated forms, such as APOE ε4-linked AD. The DSH does not claim that all sporadic AD cases are EI-AD; rather, we propose that in environmentally exposed populations—increasingly the majority in the *Plasticene* era—NPs act as a sufficient and potent initiator. Other etiologies (genetic, metal-mediated, infectious) likely coexist and may predominate in some individuals or historical cohorts. Its identification shifts the diagnostic focus toward exposure history and the detection of the synthetic component within classic lesions—a task for which standard neuropathology, focused on biological stains, is currently blind.

We emphasize that the presence of synthetic NPs at the physical center of Aβ plaques and tau tangles in human AD brain tissue is currently a prediction of the DSH, not an established finding. Direct empirical demonstration using cryo-preserved, solvent-free tissue preparations and advanced spectroscopic techniques (Raman, FTIR, py-GC/MS) is required to validate this core prediction. The DSH is offered as a testable hypothesis—one that generates specific, falsifiable predictions—not as a proven etiological model.

## 6. Diagnostic, therapeutic, and methodological implications

The DSH shifts the etiological focus of AD from inherent protein toxicity to a failed host response to an environmental trigger. This paradigm shift requires a fundamental reassessment of diagnostic criteria, therapeutic strategies, and the methodological toolkit of neuropathology itself.

### 6.1. A new diagnostic paradigm: imaging and detecting the synthetic core

If the synthetic core is central to etiology, its detection must be central to diagnosis. This requires expanding our biomarker arsenal beyond Aβ and tau. In vivo imaging requires developing novel PET ligands that target synthetic polymer surfaces, their unique adsorbed toxicant corona, or specific host-response proteins induced by NP exposure. Biofluid analysis should use advanced mass spectrometry techniques, such as pyrolysis-gas chromatography/mass spectrometry (py-GC/MS), applied to cerebrospinal fluid or blood to detect polymer-specific degradation products or characteristic pollutant profiles associated with plastic exposure. Clinically, this framework calls for systematically incorporating detailed environmental, occupational, and residential exposure histories into evaluations to identify individuals at high risk for EI-AD.

### 6.2. Novel therapeutic strategies

The DSH dictates a multi-pronged, sequential therapeutic strategy that targets the entire incursion lifecycle, moving beyond monolithic protein clearance. The most effective intervention is primary prevention: preventing neural incursion through aggressive policies to reduce non-essential plastic production and improve environmental filtration. For peripheral interception, developing "molecular sponges" such as engineered liposomes or synthetic HDL particles to bind circulating NPs in the bloodstream and promote hepatic or renal clearance could prevent CNS entry.

The ultimate therapeutic challenge is core removal or neutralization within the brain. This may involve harnessing or engineering enzymes that degrade synthetic polymers under physiological conditions, or potentiating the brain's own xenobiotic efflux pumps (e.g., ABC transporters) to export NPs, a strategy informed by research into their role in AD (Pahnke et al., 2021). These approaches must be combined with adjunctive repair of downstream damage.

This includes using NLRP3 inflammasome inhibitors to quench the pyroptotic cascade, providing metabolic support such as insulin sensitizers to restore cerebral bioenergetics, and employing niche-rejuvenating therapies to repair the exhausted stem cell and synaptic microenvironment (van Velthoven & Goldman, 2019). Preclinical evidence also supports targeting the microglial phagocytic apparatus directly: in a mouse model of AD, inhibition of protein tyrosine phosphatase 1B (PTP1B) enhanced SYK-mediated microglial clearance of Aβ, suggesting that restoring frustrated microglial function—rather than merely dissolving the proteinaceous sarcophagus—may constitute a complementary therapeutic avenue (Cen et al., 2026).

### 6.3. Validating the DSH: a forensic toolkit for environmental neuropathology

To test the hypothesis and advance the field, neuropathology must embrace a forensic materials science approach. This requires adopting techniques that avoid destructive processing. Raman microspectroscopy enables non-destructive, in situ molecular "fingerprinting" to identify specific polymers within a tissue section and map their distribution relative to Aβ plaques or tau tangles. Pyrolysis-gas chromatography/mass spectrometry (py-GC/MS) provides definitive chemical analysis of micro-dissected plaque-rich tissue, offering quantitative proof of plastic composition. Cryogenic-electron microscopy with energy-dispersive X-ray spectroscopy (cryo-EM/EDS) on cryo-preserved, unfixed tissue can visualize foreign particulate matter and analyze its elemental composition.

Implementing these tools requires a parallel evolution in brain banking protocols. Alongside standard formalin fixation, rapid cryopreservation must be adopted to preserve labile synthetic materials and native ultrastructure for these advanced analyses (McKenzie et al., 2024). By integrating these techniques, neuropathology can evolve from a purely biological discipline into an environmental neuropathology, capable of diagnosing both the brain's response and the foreign agent that provoked it. This shift is essential for testing the DSH and uncovering the full spectrum of exogenous contributors to neurodegenerative disease.

### 6.4. Methodological blind spots: why synthetic cores have evaded standard histopathology

A central challenge to the DSH is the apparent invisibility of synthetic cores in the century of literature since Alois Alzheimer's original description. However, a rigorous evaluation of modern laboratory protocols reveals a profound methodological bias toward biological materials that inadvertently mask or remove synthetic toxicants. Standard neuropathological techniques, while exquisitely refined for visualizing proteinaceous lesions, are chemically and physically destructive to the polymers proposed as the etiological trigger (Vizcarra et al., 2023; McKenzie et al., 2024).

#### 6.4.1. The "xylene/solvent" erasure and thermal degradation

The cornerstone of diagnostic neuropathology remains the formalin-fixed, paraffin-embedded (FFPE) section. As noted by Jellinger (2020), the Alzheimer's continuum is a complex mixed proteinopathy, yet our primary tools for visualizing this complexity rely on aggressive chemical clearing. To achieve micrometer-thin sections for microscopy, tissue must be dehydrated in graded alcohols, followed by "clearing" in organic solvents such as xylene or chloroform. These chemicals are potent industrial solvents for the very polymers proposed as the etiological trigger. Specifically, polystyrene and polyethylene—two of the most prevalent environmental microplastics—are highly susceptible to dissolution or structural leaching during these stages.

By the time a slide reaches the microscope, the synthetic core may have been chemically evacuated, leaving behind a "ghost" space or a hollowed-out proteinaceous shell that appears purely biological to the observer. This methodological artifact is further compounded by the paraffin infiltration process, which requires sustained heating to approximately 60°C. This temperature exceeds the glass transition or softening points of several common nanoplastic variants, potentially deforming or degrading the particles' native morphology beyond recognition. Consequently, the standard histological pipeline may have inadvertently "cleaned" evidence of the *Plasticene* trigger for over a century.

We explicitly call for systematic, side-by-side tissue studies comparing FFPE-processed samples versus matched cryo-preserved, solvent-free samples from the same AD brains. Such studies would directly test the hypothesis that standard histopathology erases synthetic evidence. If FFPE samples show no detectable plastic signal while cryo-preserved samples from the same region reveal polymer cores within plaques, this would validate the methodological blind spot and revolutionize archival tissue interpretation. To date, no such systematic comparison has been published; this represents a priority research direction.

#### 6.4.2. Visual limitations: the "translucent core" problem

Standard diagnostic stains are optimized for biological structures. Congo red and thioflavin-S bind β-sheet structures in amyloid, and immunohistochemical dyes target specific protein epitopes (Walker, 2020). A nanoplastic particle at the center of an Aβ scaffold appears as a translucent, non-staining void under standard brightfield or fluorescence microscopy—optically invisible against the background. Consequently, the synthetic core of a plaque is systematically erased or masked, while its biological shell is exquisitely detailed.

#### 6.4.3. Re-interpreting "ghost plaques" and ARIA through the DSH lens

This methodological blind spot offers a new lens for puzzling observations. The "ghost plaques" observed post-immunotherapy or in archival tissue may be empty protein shells from which the synthetic core has been dissolved during processing or treatment. Furthermore, as Lassmann (2022) notes, neuroinflammation is highly context-dependent. The DSH posits that anti-Aβ antibodies successfully remove the sarcophagus but leave behind the naked, insoluble synthetic core. Re-exposure of this toxicant to microglia would trigger a massive inflammatory rebound clinically recognized as ARIA, directly linking the therapeutic mechanism to a major side effect.

#### 6.4.4. A forensic toolkit for environmental neuropathology

Validating the DSH requires neuropathology to adopt a forensic, materials-science approach, leveraging techniques that bypass the destructive limitations of traditional organic solvents:

- Vibrational Spectroscopy (Raman/FTIR): These techniques allow for in situ molecular "fingerprinting." Raman microspectroscopy is particularly valuable for identifying specific polymers (e.g., PS, PE, polypropylene) within a tissue section at sub-micron resolution without chemical clearing.
- Pyrolysis-Gas Chromatography/Mass Spectrometry (Py-GC/MS): This remains the "gold standard" for definitive chemical identification. By applying Py-GC/MS to micro-dissected, plaque-rich tissue samples, researchers can achieve quantitative proof of plastic composition and identify the "Trojan Horse" co-pollutants adsorbed to the core. This technique also enables longitudinal exposure-disease correlation studies, which are currently lacking.
- Cryogenic Preservation and Microscopy: To avoid the thermal and chemical degradation of the FFPE pipeline, implementing parallel cryopreservation protocols is essential. Analysis by cryo-EM/EDS (Energy-Dispersive X-ray Spectroscopy) allows for the visualization of foreign particulate matter while simultaneously mapping its elemental composition (e.g., detecting the heavy metal corona) in its native state (McKenzie et al., 2024).
- Polarized Light Microscopy: As a low-cost screening tool, polarized light can detect the birefringence characteristic of many semi-crystalline polymers. This may allow for the identification of remnant synthetic seeds in archival slides where the proteinaceous "sarcophagus" has been partially degraded, but the core remains optically active.

In conclusion, the DSH does not fault past practice but reveals its limitations in addressing a novel etiological agent. By acknowledging this blind spot and expanding its technical repertoire, neuropathology can evolve into an environmental neuropathology capable of diagnosing both the brain's response and the foreign agent that provoked it. This shift is essential for testing the DSH and uncovering the full spectrum of exogenous contributors to neurodegenerative disease.

## 7. Summary and future directions

This clinicopathological update has synthesized the paradoxical failure of protein-targeting therapies with the emergent reality of pervasive neural contamination by NPs into the DSH. We have suggested reframing amyloid-β plaques and tau neurofibrillary tangles not as intrinsic pathogens but as fossilized remnants of overwhelmed, evolutionarily conserved sequestration mechanisms—an extracellular sarcophagus and an intracellular lockbox. The novel trigger of the *Plasticene* era is the indestructible synthetic polymer, which hijacks this response, forming permanent, inflammatory complexes.

The resulting state of immune frustration culminates in a glutamate-driven phase transition to microglial pyroptosis. This lytic cell death liberates synthetic seeds, propagating pathology via the glymphatic system and offering an explanation for the stereotypical spread of Braak stages. This framework coherently explains therapeutic failure, ARIA, and the metabolic crisis of AD, while pointing to a primary environmental etiology.

The DSH is presented as a testable hypothesis, not as a proven etiological model. We acknowledge the following limitations and alternative interpretations: (1) Most current evidence linking nanoplastics to AD is correlational or derived from experimental models using concentrations that may exceed typical human exposure; (2) the core prediction—nanoplastic cores within human Aβ plaques and tau tangles—has not yet been empirically demonstrated; (3) historical and contemporary AD cases likely have diverse etiologies, including genetic mutations, heavy metals, chronic infections, and other environmental toxicants; (4) intrinsic aging mechanisms may be sufficient to produce AD pathology in some individuals without significant environmental triggers; and (5) the argument that FFPE processing erases synthetic evidence remains a logical deduction until confirmed by systematic side-by-side tissue studies.

### 7.1 Critical research questions

1. Detection: Are NPs physically present within the core of Aβ plaques and tau tangles in human AD brains? The DSH predicts that cryo-preserved, solvent-free tissue preparation will reveal synthetic cores within classical lesions—a hypothesis awaiting direct testing via Raman microspectroscopy, py-GC/MS, and cryo-EM/EDS. Do they co-localize with the progression of Braak stages? Systematic side-by-side FFPE versus cryo-preserved comparisons are urgently needed.
2. Epidemiology: Can longitudinal studies correlate quantitative measures of lifetime plastic particulate exposure with AD risk, pathological burden, and specific clinicopathological subtypes? Current evidence is largely inferential; direct £prospective studies are lacking. Proposed comparative cohorts: coastal versus inland residents, military personnel (>10 years service) versus civilians, industrial versus developing nations, plant-based versus meat-based diets.
3. Mechanism: Do NP-protein complexes directly disrupt key neuronal phosphatase systems in vivo? Does controlled NP exposure in animal models recapitulate the full cascade from sequestration and immune frustration to pyroptotic spread and cognitive decline? Dose-relevance questions remain: do chronic low-dose exposures over decades produce equivalent pathology to acute high-dose experimental models?
4. Differential Susceptibility: What genetic (APOE4, ABCA7, TREM2), physiological (BBB integrity, glymphatic function), and psychosocial (chronic stress, socioeconomic status) factors determine which exposed individuals develop EI-AD or remain cognitively healthy?

### 7.2 A call for interdisciplinary collaboration

Solving EI-AD dismantles academic silos. It demands an unprecedented alliance of neuropathologists to refine detection methods; environmental toxicologists to characterize exposure and transport; polymer chemists and materials scientists to understand degradation; and public health experts and policymakers to implement prevention. In this view, the brain is the canary in the coal mine for the *Plasticene*. By confronting the synthetic sequestrum within the Alzheimer's brain, we address not only a neurological tragedy but also a profound symptom of our modern material condition. The path forward is clear: we must learn to see what we have been systematically erasing and, in doing so, redefine the disease.

## Data availability

No original datasets were generated or analyzed during the preparation of this hypothesis article. All discussed evidence is sourced from previously published, publicly available literature.

## Author contributions

MASG: Conceptualization, investigation, writing original draft, writing review and editing. The author conceived the dual sequestration hypothesis, conducted the literature synthesis, and wrote the entire manuscript.

## AI contribution

During the preparation of this manuscript, the author used large language models (Deepseek, Gemini, and Claude) for language refinement, grammar checking, and assistance with literature synthesis in response to reviewer comments. Prior to submission, Deepseek and Gemini functioned as critic-reviewers pointing out shortcomings or gaps in earlier drafts. The author reviewed and edited all AI-generated suggestions and assumes full responsibility for the final content. Gemini 3 created each of the figures in this article, which were described and refined by the author.

## Conflict of interest statement

The author, Michael A. S. Guth, declares no financial or commercial conflicts of interest. In alignment with the ethos of Free Neuropathology, the author affirms that this work was conducted in total intellectual independence. There has been no influence, funding, or oversight from pharmaceutical entities involved in amyloid/tau-clearing therapeutics, nor from industries associated with polymer production or environmental remediation. This manuscript serves solely as an independent synthesis of the published evidence, unencumbered by the institutional or corporate biases that frequently govern neurodegenerative research.
